# Understanding Adolescents’ Categorisation of Animal Species

**DOI:** 10.3390/ani7090065

**Published:** 2017-08-30

**Authors:** Melanie Connor, Alistair B. Lawrence

**Affiliations:** 1 SRUC, West Mains Road, Edinburgh EH9 3 JG, UK; 2 Roslin Institute, University of Edinburgh, Penicuik EH25 9RG, UK; alistair.lawrence@sruc.ac.uk

**Keywords:** perception, categorization, animals, card sorting, animal welfare, adolescents

## Abstract

**Simple Summary:**

When people try to make sense of the world they often use categorisations, which are seen as a basic function of human cognition. People use specific attributes to categorise animals with young children using mostly visual cues like number of legs, whereas adults use more comprehensive attributes such as the habitat that the animal lives in. The aim of the present study was to investigate how adolescents categorise different types of animals. A card sorting exercise in combination with a survey questionnaire was implemented. Adolescents were asked to group images of a variety of common British farm, pet, and wild animals that were printed on cards. Furthermore, adolescents were asked to rate a number of animals regarding their utility, likability, and fear, which served as affective responses. Results show that adolescents primarily use an animal’s perceived utility as a means for their categorisation along with their affective feelings towards those animals. In other words, adolescents group animals into farm, pet, and wild animals with one exception, birds. Birds, regardless of their role in society (pet, farm, or wild animal), were mostly grouped together. The results are important to understand adolescents’ perception of animals, which may explain the different attitudes and behaviours towards animals.

**Abstract:**

Categorisations are a means of investigating cognitive maps. The present study, for the first time, investigates adolescents’ spontaneous categorisation of 34 animal species. Furthermore, explicit evaluations of 16 selected animals in terms of their perceived utility and likeability were analysed. 105 British adolescents, 54% female, mean age 14.5 (SD = 1.6) participated in the study. Results of multidimensional scaling (MDS) techniques indicate 3-dimensional data representation regardless of gender or age. Property fittings show that affect and perceived utility of animals explain two of the MDS dimensions, and hence partly explain adolescents’ categorisation. Additionally, hierarchical cluster analyses show a differentiation between farm animals, birds, pet animals, and wild animals possibly explaining MDS dimension 3. The results suggest that utility perceptions predominantly underlie adolescents’ categorisations and become even more dominant in older adolescents, which potentially has an influence on attitudes to animals with implications for animal welfare, conservation, and education.

## 1. Introduction

People generally make sense of the world by categorising it [1]. Categorisation is a basic function of human cognition and adults use categorisation as a means of knowledge organisation about objects at different levels of abstraction [2]. When categorising, adults and children may use some or all available attributes of the object [3,4]. However, those attributes may differ and it has been shown that children value some features more than others when categorising objects [3,4]. Categorisation in general starts at a very young age; experiments revealed that 3 month old children were able to categorise cars and vehicles after being habituated to both static colour images and dynamic point light displays of animals and vehicles [4]. At the age of 8 months, infants were able to discriminate between categories such as animals versus furniture [2]. With increasing age categorisations become more complex and different mental models are known to be applied when categorising animals [5]. Pre-school children have been found to categorise animals using anatomical features such as the number of legs, presence of wings, and animals’ general appearance (e.g., small/big) [5,6,7]. At the age of 9–10 years children have the means to classify animals phylogenetically [8,9]. However, it has been shown that older children include in addition to anatomical features, also behavioural (e.g., locomotion) and environmental (e.g., habitat) traits [10,11]. Categorisations of animals often depend on the context in which categorisations are investigated. For example, adult categorisation of animals as food (or non food) plays a critical role in how we think about the animal involved [12]. In the case of categorising animals as food, the act of categorisation and the framing of animals as food may shift the focus from morally relevant attributes, and therefore may change perceptions of animal’s [12].

Understanding the people’s perceptions and attitudes of animals is of relevance, especially in the field of animal welfare e.g., [13] but also in areas such as conservation [14] and education [15]. Attitude research has a long tradition in social psychology, mainly due to its relationship with human behaviour [16]. Various researchers have proposed models of attitudes in general, with one of the most commonly used being a trilogy of affect, cognition and conation [16]. In this model, affect refers to a person’s feelings towards, and evaluation of, for example, a person, an issue, an event, or an animal. These evaluations occur spontaneously and do not require a thorough evaluation of the object [17,18]; cognition refers to the knowledge, beliefs, and thoughts about the object; and, conation refers to behavioural intentions and actions with respect to the object [16]. With regard to attitude formation towards animals, it has also been postulated that there are three fundamental motivational forces, which may well provide a foundation for understanding the human-animal relationship [19]. In this specific model, attitudes depend partly on people’s experience of animals. However, the instrumentality or usefulness of animals has tended to be the dominant dimension in this regard [19]. Perceptions of animals’ usefulness have been shown to differ between male and females [20,21]. Furthermore, men scored higher on environmental and scientific attributes of animals, whereas women scored higher on ethical and moralistic attributes [21]. More recently, Serpell [13] proposed that in addition to utility affect may also play an important role when forming attitudes to animals. Utility in this model represents people’s perceptions of animals’ instrumental values and affect representing affective or emotional responses to animals [13]. In this model, affect and utility are presented as continua between positive and negative poles in a two-dimensional space with any animal or organism being represented in this two-dimensional space depending on its perceived utility and the affect it evokes [13]. Interpreting the earlier work of Kellert [20], using Serpell’s [13] two-dimensional framework suggests that men may be more relying on their utility judgements of animals, whereas women tend to be more influenced by the affective dimension when forming their attitudes towards animals. Other studies have found that males had less concern for animal welfare, both in self reported tendency to take action to help members of other species, and in sensitivity to their use by humans [22]. Women were less comfortable than men with animals having more negative reputations such as spiders, snakes, and toads [22]. As yet there has been no empirical study directed at testing Serpell’s [13] framework.

When investigating attitudes towards animals using the Pet Pest Profit animal attitude scale, pets were liked best [23]. Research often distinguishes between different classes and species of animals. As a consequence, the development of attitudes towards pet animals has been well investigated. However, other categories of animals have not been included. A study investigating the link between the bio-behavioural similarity between humans and given animal species found that humans have more positive attitudes towards species on the basis of shared bio-behavioural traits [24]. Bio-behavioural similarities between humans and animals were based on factors such as size, weight, lifespan, reproductive strategies, parental investment, and social organisation. Animals which are perceived to share a lot of traits with humans, were liked more than animals that only share some or none of the traits with humans [24].

### 1.1. Studying Categorisations

Different methodological approaches can be employed to investigate people’s perceptions or categorisations. Qualitative interviews e.g., [7] as well as quantitative questionnaires e.g., [11] have been used to investigate children’s categorisations of animal species. Each methodology poses its own constraints; qualitative methodologies rely heavily on researcher’s subjective interpretations, which subsequently influenced data interpretation [25]. Quantitative methodologies, such as survey questionnaires, account for subjectively interpreted data by obtaining responses, which directly translate into quantitative terms and do not require further subjective interpretation [25]. However, it has been shown that people use pre-defined criteria, construct their preferences while answering questionnaire questions, and not only rely on their knowledge but also other information available to them, such as questions in the questionnaire [26]. It is therefore favourable to implement the methodologies that do not lead participants into a pre-defined direction and allow for a greater variety in answers. Such methodologies comprise conjoint tasks and similarity ratings. Conjoint tasks are mainly utilized in market research to evaluate consumer preferences [27]. Similarity, ratings often facilitated through card sorting have been implemented in various disciplines and allow for the investigation of participants’ mental representations or perceptions and have been employed studying human perceptions of animals [10,24,28]. Card sorting has a long history in social science studies due to a variety of advantages, including the ease of administration, low susceptibility to experimental demand characteristics, and economy in handling large numbers of objects or stimuli (Whaley, 2009). A differentiation has been made between free card sorting—non-restricted sorting of cards and card sorting- restricted sorting of cards [29] e.g., providing specific/detailed instructions. Card-sorting techniques are usually analysed by using Multi-dimensional-Scaling (MDS), which refers to a class of scaling techniques that convert a matrix of proximities into a geometric configuration or a map of points in a multi-dimensional space [30,31].

### 1.2. Rationale of the Present Study

Attitudes towards animals are gaining importance for a wide range of issues including animal welfare, conservation, and sustainable consumer behaviour. It has been shown that attitudes towards animals develop early in people’s life [32], however most studies have been conducted with kindergarten or primary school aged children [6,11,32]. Furthermore, the adult attitudes towards animals seem to be greatly influenced by childhood experiences [33], and adult perceptions of animals have been hypothesised to vary depending on perceived utility and affect [13]. However, there is a lack of studies using adolescent samples. Adolescence is a crucial time in people’s lives with considerable changes in the social and affective processing abilities [34]. These changes in social-affective processing may confer adaptive changes, such as a greater flexibility in adjusting to intrinsic motivations and priorities amidst changing social contexts in adolescence [34]. The present study will therefore investigate adolescents’ spontaneous perceptions and categorisations of animals by means of a free card sorting technique. Sorting techniques are an effective way to investigate how much agreement and disagreement there is between respondents regarding a pre-defined topic or area [35]. Free card sorting is also a useful technique to identify relevant categorisation without providing dimensions to be used for categorising. Card sorting techniques are spontaneous and quick, they are easy to apply, and are systematic and they facilitate investigating affective evaluations of a given subject. Card sorting has been shown to be the preferred technique over pairwise ratings or similarity ratings due to it being more natural, interesting, and comprehensible than those techniques [36]. Card sorting techniques are widely used in the studies of knowledge acquisition [35], and have been successfully implemented to investigate people’s categorisation of different biotechnology applications [37]. Furthermore, the present study aims to understand what underlies adolescents’ categorisation of animals. Therefore, the theoretically hypothesised dimensions of affect and utility [13] will be tested by using statistical property fitting. The results of the present study will be discussed with regard to Serpell’s [13] theoretical assumptions and their implications for animal welfare, but also in a wider environmental context. 

## 2. Methods

### 2.1. Card Sorting Task

A card-sorting task using picture cards was conducted by means of a face-to-face interview. Each participant was interviewed individually in a different room during class time. Interviews were prior agreed with the head teacher and teacher of the relevant class, and written consent was gained from parents, explaining the purpose of the study and that data will not allow for identifying neither the school that pupils attend, nor the pupils themselves. At the beginning of the interview, demographic data were obtained from the participants including, age, gender, and pet ownership. Afterwards participants received a shuffled set of 34 cards with each card having an animal print on it. Animal pictures came from a set of animal pictures provided online by Sparklebox (www.sparklebox.co.uk), an online provider of educational materials. Animal pictures were coloured drawings of animals with a blank background ensuring that there were no distractions with the animals being illustrated in a neutral state. Animals comprised of British farm, wild, and pet animals (Table 1). Participants were asked to familiarise themselves with the cards, and were allowed to ask the name of the animal if it was unknown to them. Subsequently, participants were asked to sort the cards into between 1 and 33 categories. There were no further instructions as to how to categorise the animals. However, each animal could only be assigned to one category, and all animals had to be sorted. There was no time limit imposed. After adolescents completed the sorting task, they were asked to describe and name the piles they had created.

After completing the card-sorting task, participants were asked to fill out a short questionnaire for the purpose of which sixteen animals from the card-sorting task were selected. These animals are designated with a * in Table 1, with attention taken to ensure that farm, wild, and pet animals were included in the questionnaire assessment. Participants were asked to assess: likeability (positive affect) by being asked how that much they liked the selected animals; how much they feared the animal (negative affect); and, utility as to how useful they regarded the selected animals. Each construct was rated on a 6 point-likert type scale ranging from 1 = not liked at all/no use at all/not feared at all to 6 = liked very much/very useful/feared very much. 

The card sorting task gained ethical approval by the ethics committee of the University of St. Andrews a collaborating institution for this DEFRA funded study. 

### 2.2. Data Analysis

Multidimensional scaling techniques (MDS) were utilised to investigate the structure (visual representation) of the data in IBM SPSS Statistics 22. MDS are a useful means with which to explore relationships within a data set. Specifically, distances between data points are investigated with the distances in space matching the similarities of the data as closely as possible. In this case, animals that have been categorised together will have higher similarity ratings (higher number of occurrences in one category) than animals that have not been placed into the same category so often. MDS for individual differences (INDSCAL) was applied to investigate the sub-groups (males, females, and different age groups). INDSCAL allows for the investigation of individual task matrices, and therefore achieves a unique orientation of the coordinate axes; a metric inference is made to connect the similarities to the distances [38]. A hierarchical cluster analysis was employed to investigate the hierarchical structure of participants’ categorisations. Furthermore, property fitting was applied to investigate the theoretical assumption that the participants’ attitudes towards the animals were determined by affect and utility. Qualitative content analysis and frequencies were used to analyse the categories named by the adolescents.

### 2.3. Participants

In total, 105 Scottish adolescents participated in the study. Data were collected in 7 different schools, including 2 inner city schools, 2 private schools, 1 academy, 1 rural secondary school in an affluent area, and 1 rural secondary school in a deprived area (due to anonymity and ethical guidelines schools can not be further identified). All participants attended their age appropriate class and no control measures were applied for adolescents’ academic performances. 54 (51.4%) were female and 51 (48.6%) were male. The mean age was 14.48 years (standard deviation (SD) = 1.58), and most N = 88 (83.8%) adolescents reported to have a pet in their home. 45 (42.86%) adolescents reported to live in towns, 33 (31.43%) reported to live in cities, and 27 (25.71%) adolescents reported to live in rural locations.

## 3. Results

### 3.1. Card Sorting and Multi-Dimensional Scaling

On average, adolescents sorted the animals into five different categories (mean = 5.35, SD = 2.25, min = 2, max = 11). There was a significant difference between boys and girls; girls (mean = 4.93, SD = 1.87) created fewer groups than boys (mean = 5.80, SD = 2.53, *t* (91.9) = −2.03, *p* = 0.047, Cohen’s d = 0.391). 

In order to test whether the males and females also differ in their mental representation of the animals, Stress-I values were used to describe the goodness of fit and were for the present study: 0.41, 0.21, 0.11, 0.08, and 0.07 for dimensions 1 to 5, respectively. A decision was made to stop at 5 dimensions, as Stress-I values did not significantly decrease when including further dimensions [38]. The Stress-I value of 0.11 for the three dimensional solution and the interpretability of the three dimensions implied selecting the three-dimensional solution. A comparison of dimension weights indicated no differences between the male and female participants. The dimension weights for the first three dimensions were: dimension one: male = 0.405 and female = 0.418; dimension two: male = 0.396, female = 0.395; dimension three: male = 0.397, female = 0.385. 

**Figure 1:**
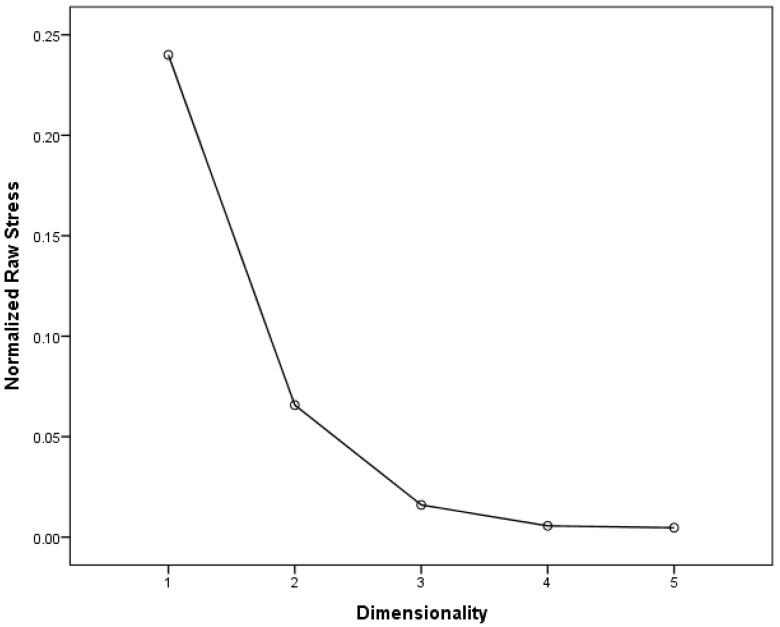
Stress-1 values for the whole sample.

**Figure 2:**
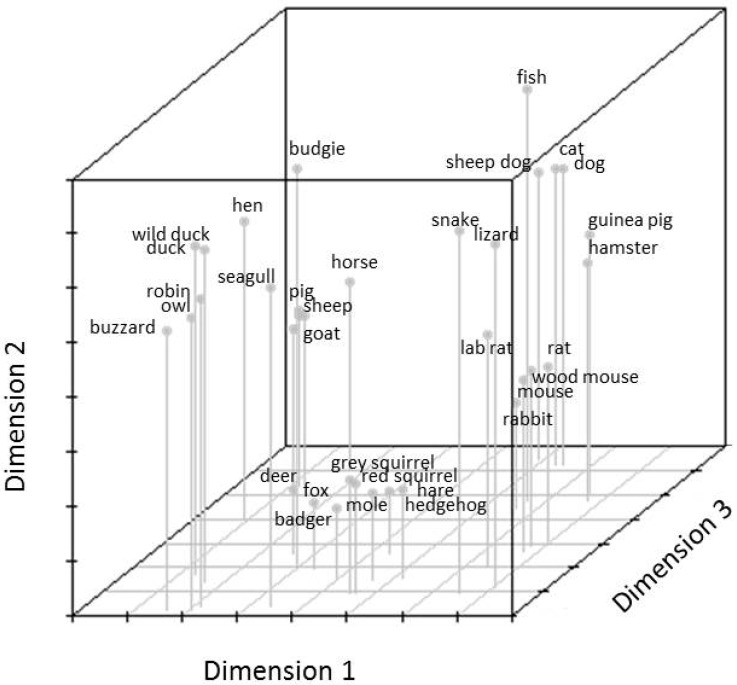
Adolescent’s mental representation of the animal species illustrated on the sorting cards. MDS solution for all adolescents together.

**Figure 3:**
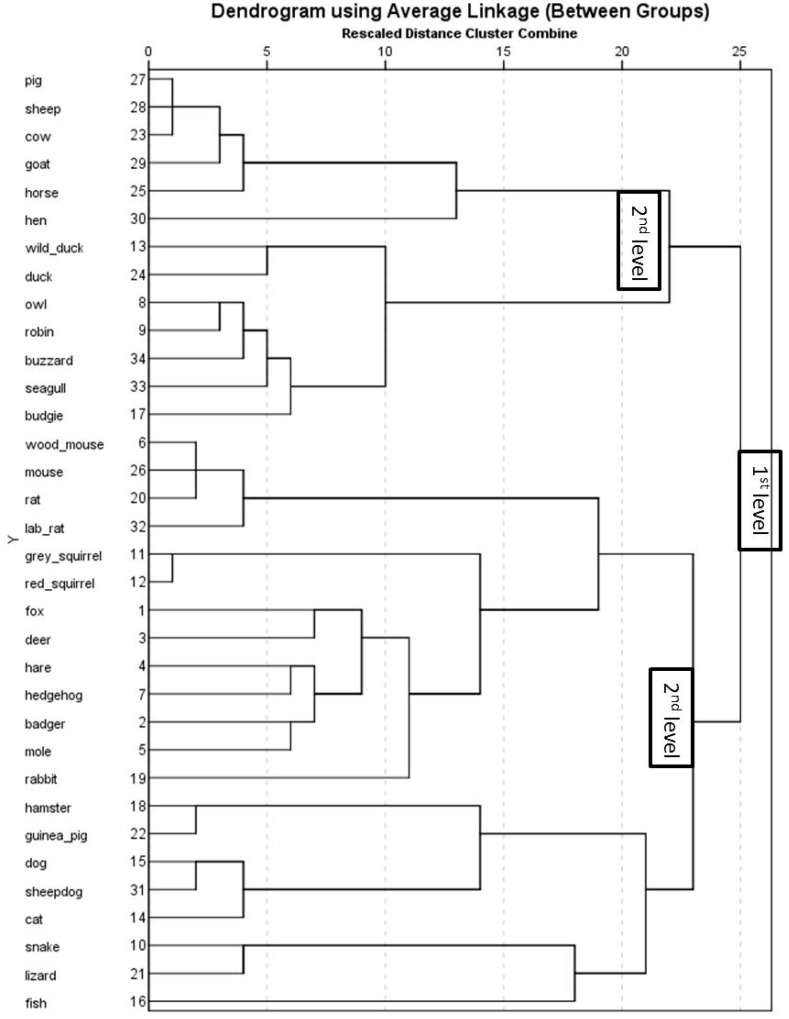
Hierarchical cluster analysis using average linkage of animal species for the whole sample.

A similarity matrix was therefore created for the whole sample and further analysed using the PROXSCAL (multidimensional scaling of proximity data) method. This method allows for investigating a least squares representation of the animals in a low-dimensional and meaningful space [39]. In order to determine the dimensionality of the data, Stress-I values were analysed and plotted in a graph together with the dimensions (Figure 1). The analysis was stopped at 5 dimensions, as again there was no further decline in Stress-I values. Stress-I values for the five-dimensional solution for the combined matrix were: 0.24, 0.07, 0.02, 0.006, and 0.005 for dimensions 1 to 5, respectively. The data were again best explained in a 3-dimensional space, indicated by an ‘elbow’ between the second and third dimension (see Figure 1), and a Stress-I value of 0.02 indicating an excellent fit [38]. The results indicate a differentiation between birds, farm animals, pet animals, and other animals (Figure 2). In order to investigate the underlying structure of the categorisations, a hierarchical cluster analysis was also performed. The results indicate that two main clusters were formed at the first level (Figure 3), with one cluster including farm animals and birds, and the other cluster including all other animals. At the second level, farm animals also form one group and birds another, with the ‘chicken’ being equidistant to either cluster. At the second level, another cluster comprised of two sub-clusters, one containing all of the animals that could easily be labeled as pet animals in the UK and the other cluster comprising of wild animals (including rats and mice). 

**Figure 4:**
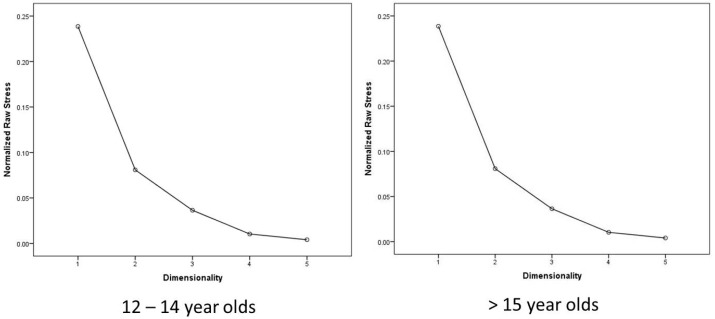
Stress-I values for the 1 to 5 dimensional solutions for both age groups.

INDSCAL was applied to investigate the differences between younger adolescents (12–14 years old, N = 53, Nfemale = 28, Nmale = 25) and older adolescents (>15 years old, N = 52, Nfemale = 26, Nmale = 26). Stress I-values were: 0.422, 0.219, 0.123, 0.088, 0.081 for dimensions 1 to 5, respectively. The Stress-I value of 0.123 of the three dimensional solution along with the interpretability of the dimensional solution again implied selecting the three dimensional solution. The comparison of the dimensional weights shows that the vectors are not identical (Table 2), and therefore, the two age groups were analysed separately using PROXSCAL, and similarly to the full sample Stress-I values indicated a three dimensional solution (Figure 4).

**Figure 5:**
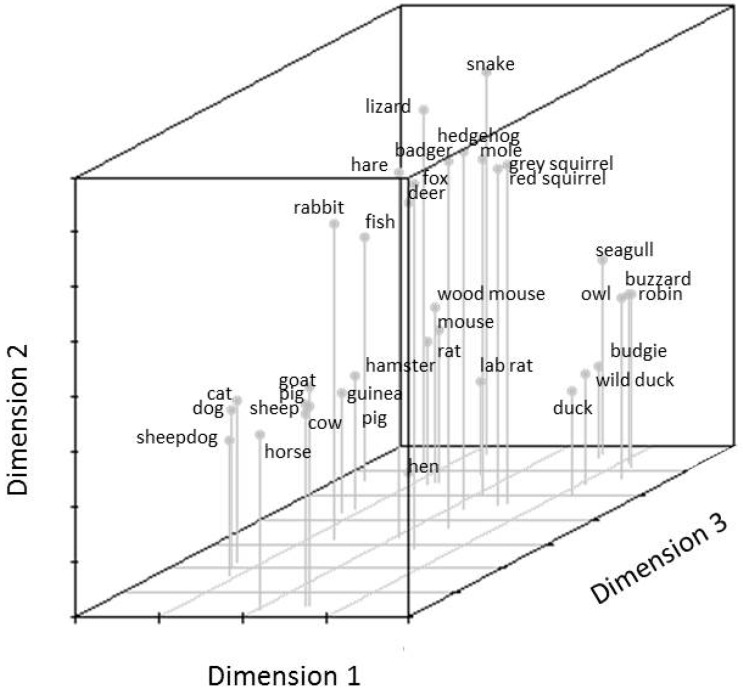
12–14 year old adolescent’s mental representation of the animal species illustrated on the sorting cards.

**Figure 6:**
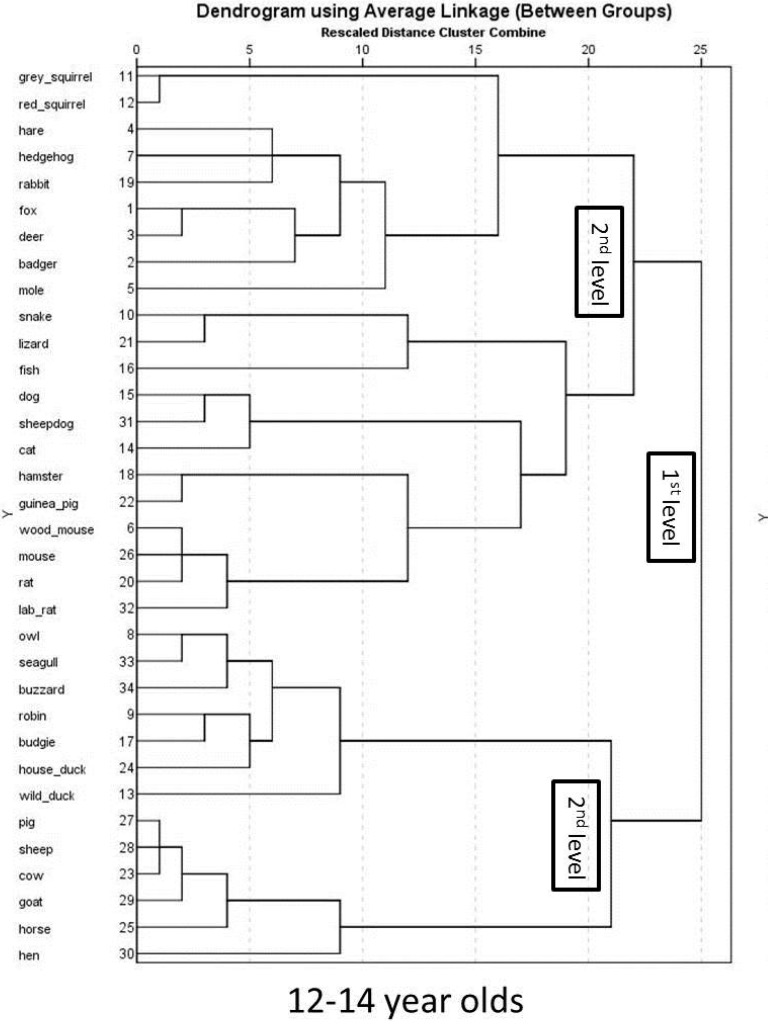
Hierarchical cluster analysis using average linkage for 12–14 year olds.

12 to 14 year olds mental representation of animals is illustrated in Figure 5. The orientation of animals within the graph differs from the whole sample but also shows a differentiation between (a) farm animals; (b) birds; (c) lizard, snake and fish and (d) all other animals. Qualitative content analysis of the species within the clusters revealed that the same animals formed the cluster of farm animals and birds when comparing 12–14 year olds with the whole sample but differ when comparing to adolescents older than 15 years. However, similar to the whole sample, 12 to 14 year old adolescents’ results of the hierarchical cluster analysis show two main clusters at the first level, the first cluster comprising two distinct sub-clusters of birds and farm animals (Figure 6). The second first level cluster also sub-divides into two sub-clusters, one comprising all wild animals (including rabbits) and the other cluster all pet animals (including mice and rats). The whole sample of adolescents included rats and mice in their category of wild animals.

**Figure 7:**
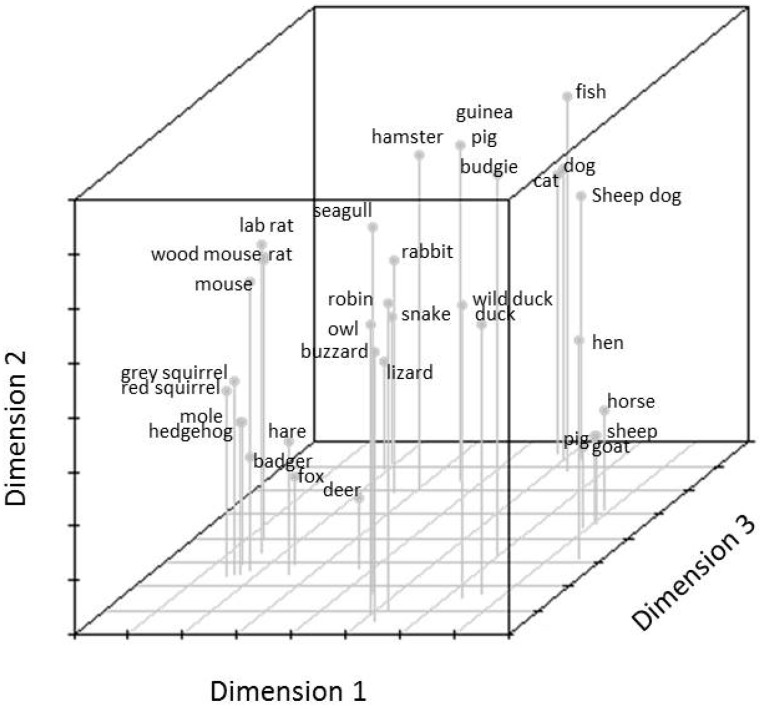
>15 year old adolescent’s mental representation of the animal species illustrated on the cards.

**Figure 8:**
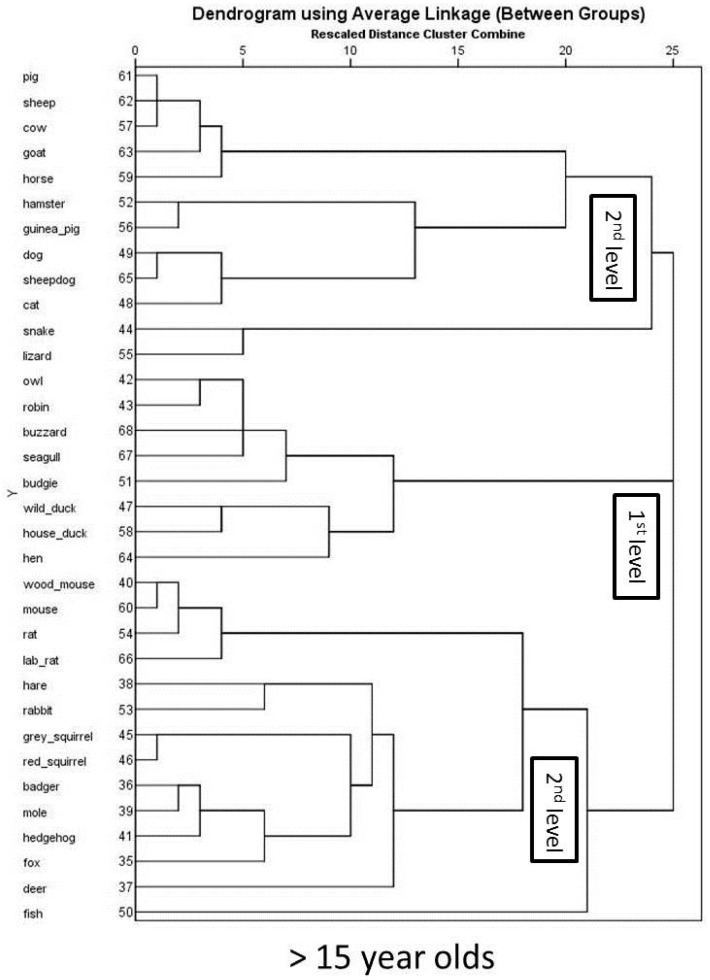
Hierarchical cluster analysis for >15 year olds, dendograms using average linkage.

Mental representations of adolescents older than 15 years old are shown in Figure 7. Again, the orientation of species in the map is different to the whole sample, and younger adolescents. Nonetheless, a differentiation between (a) pet animals; (b) farm animals; (c) birds; and, (d) wild animals is detectable. Results from the hierarchical cluster analysis of adolescents 15 years and older shows three first level clusters (Figure 8), which differs from younger adolescents and the whole sample. The first cluster comprises of three sub-clusters of pet animals (excluding mice, rats, and rabbits), exotic pets, and farm animals. The second first level cluster comprises of all the birds and the third first level cluster comprises of wild animals (including mice, rats, and rabbits). 

### 3.2. Naming of Categories

In total, 80 participants were able to name or describe the groups of cards they created. The most frequently mentioned groups were pets (*n* = 66), which included descriptions of ‘animals which people keep in their house’, ‘household animals’, and ‘house pets’; farm animals (*n* = 58), which included descriptions like ‘animals we keep in stables’, ‘farmyard animals’, and ‘animals we eat’; birds (*n* = 51), which also included the description ‘flying animals or animals which can fly’; wild animals (*n* = 47), which included the description of ‘woodland animals and forest animals’. Further frequently used concepts to describe the groups included reptiles (*n* = 26), fish (*n* = 19), rodents (*n* = 16) and small animals (*n* = 13). One adolescent sorted the animals regarding their colour, one by the presence of fur, and feathers, two adolescents used size as sorting criteria (small, medium, and big), one adolescent sorted the animals regarding his personal likes and fears, and one adolescent used the number of legs as a sorting criterion. Less frequently named groups encompassed outside of animals (*n* = 9), vermins, rats, and mice (*n* = 7), prey and predator/hunter (*n* = 3), amphibians (*n* = 3), underground animals (*n* = 2), mammals and mammals with horns (*n* = 2), common animals (*n* = 1), abstract animals (*n* = 1), and small food animals (*n* = 1).

### 3.3. Property Fitting

Participants rated 16 animals regarding their liking, disliking (fear), and the perception of the utility of each animal. Results show that adolescents liked dogs most, followed by cats, robin, and deer (Table 3). Furthermore, they expressed the most fear of foxes, followed by cows, seagulls, and buzzards. However, adolescents did not express high ratings of fear in general (Table 3). Adolescents perceived cows, chickens, dogs, and sheep as very useful (Table 3). There was a positive correlation between adolescents mean rating of utility and liking (r = 0.492, *p* < 0.001).

In order to provide an interpretation of the three-dimensional solution, a property fitting was employed [40]. A multiple regression analysis was conducted to explore the evaluative dimensions in more detail. Mean ratings of likability, fear, and utility for the selected 16 animals were used as predictor variables for each dimension. It needs to be noted that no inferences can be made to the other animals not used for the evaluation. Data were analysed for the whole sample of adolescents together, and also for the two age groups separately. The results of the whole sample show that dimension 1 could be explained by how much adolescents like the animals but also by how useful they find the animal (Table 4). Predictor variables explain 40.6% of the variance of dimension 1. Dimension 2 could clearly and solely be explained by the perceived utility of the animal. All three predictor variables explain 80.9% of the variance of dimension 2. Dimension 3 could not be explained by any of the predictor variables (Table 4). Analysing participants between 12 and 14 years of age showed that dimension 2 could be explained by adolescents’ perceived utility of the animal (Table 4). All predictor variables explain 80.6% of the variance. Dimension 3 could be explained by the adolescent’s perception of fear; 43% of the variance was explained for dimension 3. Dimension 1 could not be explained by a single predictor variable (Table 4). Results for adolescents older than 15 years show that dimension 1 can be mostly explained by the perceived utility of the animal, and to a smaller extend by perceived likeability, 69.9% of the variance is explained by all predictor variables (Table 4). Dimension 2 and 3 could not be explained by any of the predictor variables (Table 4).

## 4. Discussion

The present study investigated adolescents’ spontaneous categorisation and perceptions of a range of animal species by means of free card sorting. Employing the free card sorting technique allowed for the investigation of the adolescents’ own categories and evaluative dimensions without the adolescents being primed prior the categorisation task. Using multidimensional scaling techniques and hierarchical cluster analysis, the results show that adolescents’ mental representations of animals could best be interpreted in a three dimensional space, regardless of age or gender. Participants’ mental representations of animals, and the MDS results were graphically depicted for the two age groups (12–14 year olds, >15 year olds), and the whole sample separately. Results show that all of the participant groups generally categorise animals into four main categories encompassing, wild animals, farm animals, pets, and birds. It has to be noted that there are a few differences as to the level at which these categorisations take place. It seems that adolescents’ categorisation of animals can be explained in multiple ways. It is obvious that adolescents, regardless of age and gender, distinguish between pet, farm, wild animals, and birds. When asking adolescents to name their categories, these four animal groups were frequently mentioned. Results of the hierarchical cluster analysis show that adolescents older than 15 years spontaneously categorise animals into three main categories: (1) pet animals (e.g., dog, cat), exotic pet animals (snake and lizard, which could also be seen as wild animals), and farm animals; (2) birds; (3) wild animals (including rats, mice and rabbits). Whereas, adolescents younger that 15 years show two main first level clusters; (1) farm animals and birds; (2) pet animals and wild animals, which at the second level divide into (a) farm animals; (b) birds; (c) pet animals; and, (d) wild animals. However, the species belonging to each category and subsequent sub-categories can vary, which is especially true for rats, mice, and rabbits. Younger adolescents (12–14 years old) categorised mice and rats together with guinea pigs and hamsters, which corresponds to their phylogenetic order (rodents). It has been shown that children are able to classify animals hierarchically from about 9 to 10 years old [8,9]. However, older adolescents (>15 years) categorised mice and rats together with wild animals, possibly using habitat as a categorisation dimension. In general, adolescents’ categorisation of animals could be interpreted as a function of habitat (farm, domestic, wild), locomotion (flying, moving on land), or a function of use (pet, farm, wild animals), which has been shown in other studies too [11,20]. Adolescents’ descriptions of their categories show that a mixture of criteria was applied to categorise the species, they rarely used just one single criterion. Most adolescents use habitat or use together with locomotion (flying), or a biological taxonomy (birds). Birds were the only animal class for which adolescents used the biological taxonomy frequently. Very few adolescents mentioned reptiles and mammals. This, however, doesn’t necessarily imply that adolescents do not know how to classify animals biologically, it maybe suggests that taxonomy is not a criterion adolescents use spontaneously. 

In addition to the card-sorting task, adolescents filled out a short questionnaire that asked them to rate a selection of the species based on perceived utility, likeability, and fear felt. Likeability and fear were intended to reflect opposing emotional responses to the various species. Results show that farm animals are perceived as being very useful, followed by pet animals and wild animals, which corresponds with Kellert’s utilitarian attitude dimension, and represents a primary concern for the perceived practical and material value of animals or the animals’ habitat [20]. Adolescents reported to like dogs and cats the most, which supports the literature of positive attitudes and attachment to pet animals [32,39]. These positive attitudes could be a result of the high level of pet ownership in the present sample. In the present study, more that 80% of the adolescents reported to have pets at home, which is in line with the general UK population [41]. Therefore, it is not surprising that dogs and cats received the highest ratings for likability. Furthermore, dogs and cats are often regarded as family members and share a lot of bio-behavioural traits with humans. Such as parental investment or reproductive strategies. It has been shown that humans like species on the basis of these shared bio-behavioural traits [24]. Despite adolescents showing generally high ratings for liking of animals, seagulls and grey squirrels are among the least liked animals, possibly due to them being perceived as nuisance species. 

The ratings of likability and utility perceptions were used to investigate the underlying dimensions of adolescents’ categorisations with likability being used as a proxy for affect. Results of the property fitting show that the perceived utility and likeability of animals could explain two dimensions; the third dimension could not be explained by the investigated predictor variables. Our results are in line with Serpell’s [13] theoretical model of attitudes emerging from the interaction of perceived utility and affect of the species in question, as shown for adult populations by using different methodologies (e.g., rating scales) [21]. In the current study, the perceived utility of animals seems to be a main contributor of how adolescents categorise animals (utility explains 81% of the variance of dimension 2 for the whole sample). Perceived utility has previously been suggested to be the dominant concern for developing attitudes towards animals in adults [19]. Whereas, children’s attitudes seem to be a result of pet ownership and attachment to pets [42]. There are differences between younger and older adolescents about which dimension is explained by perceived utility, and which dimension is explained by likability. Younger adolescents also use fear, which represents a negative affect to categorise animals. Furthermore, results of the whole sample show an overlap of likeability and utility. Additionally, a medium strong correlation between likeability and utility was present. Likeability of an animal was used as a proxy of affect, and may not be the most accurate measure of affect. Nevertheless, this correlation and the result that for some dimensions likeability and utility are significant predictors, indicate that the perception of animals in adolescents depends upon both likeability and utility. However, the direction or causality of the relationship can not be determined with the current dataset. It has to be noted that we found that the explained variance for likeability decreases with increasing age. This could mean that older adolescents may rely more on their utility perceptions when categorising animals than younger adolescents. Results of the present study indicate a shift in attitude formation during adolescent years, from attachment and affection towards pets, to the perception of utility, which in turn is of great importance especially in the fields of animal welfare [13], conservation [14], and education [15].

In contrast to Serpell’s [13] model, adolescents seem to also use a third dimension to categorise animals which can not be explained by the predictor variables used. This dimension most likely reflects the categorisation into farm animals, pet animals, birds, and wild animals.

## 5. Conclusions

The present study investigated the underlying determinants of adolescents’ categorisation of animals. We found that participants categorised animal species using a variety of criteria, including biological and environmental characteristics, but also used the affect-utility framework as proposed by Serpell [13]. However, the shift in older adolescents (>15 years) to use perceived utility as a main criterion to categorise animals suggests a developmental change in perceptions of animals to a more objectified perception of animals. Similar results have been shown investigating adults [43]. Perceptions and understanding of animals’ utility may therefore develop during mid to late adolescence. Considering this shift in perceptions of animals, the present study also concludes that adolescent years are a crucial time for the development of attitudes towards animals with long lasting effects and implications for animal welfare, conservation, and education.

## Figures and Tables

**Table 1:** Animals illustrated on the sorting cards (animals marked with an * were used for the property fitting).

Wild Animals		Pet Animals	Farm Animals
Fox *	Barn owl *	Cat *	Cow *
Badger	Robin *	Dog *	Duck
Deer *	Mallard duck	Goldfish	Horse
Hare	Seagull *	Budgie *	Pig *
Mole	Buzzard *	Hamster	Sheep *
Wood mouse		Rabbit	Goat
Hedgehog *		Rat	Chicken *
Grass snake		Lizard *	Sheep dog
Grey squirrel *		Mouse	
Red squirrel		Guinea pig Lab-rat	

**Table 2:** Dimension weights of the INDSCAL solution for the two different age groups.

Group	N	Dimension 1	Dimension 2	Dimension 3
12–14 year olds	53	0.384	0.403	0.410
>15 year olds	52	0.431	0.389	0.376

**Table 3:** Mean and standard deviation (SD) for adolescent’s perceptions of liking, fearfulness and utility (scale 1 = not at all, 6 = very much).

Animal	Like		Fear		Use	
	Mean	SD	Mean	SD	Mean	SD
fox	3.70	1.32	2.39	1.35	2.61	1.22
barn owl	4.01	1.24	1.72	1.04	3.00	1.23
cat	4.38	1.65	1.58	1.15	4.28	1.40
cow	3.87	1.14	2.06	1.14	5.43	0.92
deer	4.26	1.21	2.00	1.19	3.83	1.37
robin	4.36	1.37	1.23	0.78	2.57	1.03
dog	5.52	1.05	1.79	1.33	5.31	0.97
pig	4.05	1.24	1.72	1.01	5.12	1.13
hedgehog	4.39	1.31	1.49	1.03	2.57	1.13
seagull	2.38	1.22	2.06	1.37	1.95	1.09
budgie	3.82	1.36	1.44	1.00	2.66	1.37
sheep	4.05	1.24	1.60	0.92	5.29	0.86
grey squirrel	3.30	1.56	1.72	1.07	2.27	1.19
buzzard	3.76	1.44	2.04	1.28	2.93	1.44
lizard	4.16	1.46	1.98	1.25	2.50	1.31
chicken	4.12	1.28	1.54	1.02	5.34	0.89

**Table 4:** Multiple regression analysis (property fitting) for the whole sample, 12–14 year olds and >15 year olds.

Predictors	All Adolescents Together						12–14 Year Olds						>15 Year Olds					
	**Dimension 1**		**Dimension 2**		**Dimension 3**		**Dimension 1**		**Dimension 2**		**Dimension 3**		**Dimension 1**		**Dimension 2**		**Dimension 3**	
		*p*		*p*		*p*		*p*		*p*		*p*		*p*		*p*		*p*
Likeability	0.259	0.042	0.082	0.606	−0.048	0.888	−0.574	0.083	0.023	0.888	−0.351	0.215	−0.488	0.028	0.397	0.180	−0.113	0.711
Utility	−0.193	0.022	0.858	0.000	−0.090	0.778	0.041	0.887	−0.902	0.000	−0.546	0.052	0.925	0.000	0.340	0.222	−0.331	0.261
Fear	0.272	0.296	0.031	0.823	−0.364	0.229	−0.226	0.407	−0.201	0.164	−0.523	0.044	−0.342	0.066	0.184	0.449	−0.451	0.105
R^2^	40.6%		80.9%		13%		27.3%		80.6%		43%		69.9%		38.3%		30.3%	
